# Determinants of distance walked during the six-minute walk test in patients undergoing cardiac surgery at hospital discharge

**DOI:** 10.1186/1749-8090-9-95

**Published:** 2014-05-31

**Authors:** Géssica Uruga Oliveira, Vitor Oliveira Carvalho, Lucas Pereira de Assis Cacau, Amaro Afrânio de Araújo Filho, Manoel Luiz de Cerqueira Neto, Walderi Monteiro da Silva, Telma Cristina Fontes Cerqueira, Valter Joviniano de Santana Filho

**Affiliations:** 1 Master’s Degree of the Postgraduation Program in Health Sciences at the Federal University of Sergipe, - UFS, Aracaju, SE, Brazil; 2 Departamento de Fisioterapia da Universidade Federal de Sergipe - UFS, Aracaju, SE, Brazil; 3 The GREAT Group (GRupo de Estudos em ATividade física), Aracaju, Brazil; 4 Curso de Fisioterapia da Universidade Tiradentes - UNIT, Aracaju, SE, Brazil; 5 Departamento de Fisioterapia da Universidade Federal de Sergipe - UFS, Lagarto, SE, Brazil

**Keywords:** Six-minute walk test, Exercise, Cardiac surgery, Physiotherapy

## Abstract

**Introduction:**

The aim of this study was to identify the determinants of distance walked in six-minute walk test (6MWD) in patients undergoing cardiac surgery at hospital discharge.

**Methods:**

The assessment was performed preoperatively and at discharge. Data from patient records were collected and measurement of the Functional Independence Measure (FIM) and the Nottingham Health Profile (NHP) were performed. The six-minute walk test (6MWT) was performed at discharge. Patients undergoing elective cardiac surgery, coronary artery bypass grafting or valve replacement were eligible. Patients older than 75 years who presented arrhythmia during the protocol, with psychiatric disorders, muscular or neurological disorders were excluded from the study.

**Results:**

Sixty patients (44.26% male, mean age 51.53 ± 13 years) were assessed. In multivariate analysis the following variables were selected: type of surgery (*P* = 0.001), duration of cardiopulmonary bypass (CPB) (*P* = 0.001), Functional Independence Measure - FIM (0.004) and body mass index - BMI (0.007) with r = 0.91 and r2 = 0.83 with *P* < 0.001. The equation derived from multivariate analysis: 6MWD = Surgery (89.42) + CPB (1.60) + MIF (2.79 ) - BMI (7.53) - 127.90.

**Conclusion:**

In this study, the determinants of 6MWD in patients undergoing cardiac surgery were: the type of surgery, CPB time, functional capacity and body mass index.

## Background

Cardiac surgery is a well-established procedure worldwide due to its safety and effectiveness in treating cardiac patients. Among cardiac surgery, we highlight the CABG and valvar replacement [1].

Despite the undeniable efficiency, recovery of functional capacity of patients in the postoperative period of cardiac surgery is one of the important aspects and discussed in literature [2]. A simple and efficient method to directly assess the functional capacity of the patient in the postoperative period of cardiac surgery is through the six-minute walk test (6MWT). To perform the test, patients are instructed to walk as far as possible in six minutes and this maxim distance walked reflects his physical ability [3,4]. It is known that the distance ≥ 300 m covered in the 6MWT is a predictor of increased survival at 5 years of follow-up of elderly patients undergoing cardiac surgery [5]. Recently, the ability or inability to perform the 6MWT in patients undergoing cardiac surgery in pre-hospital discharge was used as a predictor of risk of postoperative complications and mortality [6,7]. Nevertheless, this is still unclear in the literature the determinants factors of the distance walked in the six-minute walk test (6MWD).

Given that the use of the 6MWT is growing in population undergoing cardiac surgery, the aim of this study was to identify the determinants of 6MWD at hospital discharge in patients undergoing cardiac surgery. In addition to establishing a reference equation and test its reliability.

## Methods

### Study design

This is an observational study that aimed to investigate the determinants of 6MWD in patients undergoing cardiac surgery.

This study was performed at a tertiary cardiology hospital in Brazil. The patients’ assessments were performed in two stages: preoperatively and at discharge. Data from patients’ records were collected, spirometry and implementation of the Functional Independence Measure (FIM) questionnaire and the Nottingham Health Profile were performed. The 6MWT was performed only at the time of patient’s discharge.

### Studied population

Patients undergoing elective cardiac surgery, coronary artery bypass grafting and/or valve replacement were eligible for this study. Patients aged over 75 years; who required more than 24 hours of mechanical ventilation or surgical reintervention, patients who had arrhythmia during mechanical ventilation protocol, patients with psychiatric disorders, neurological or muscular disorders that prevented the completion of the protocol were excluded from the study. Patients were recruited from a tertiary hospital cardiology from December 2011 to August 2013. The patients’ characteristics are listed in Table 1.

**Table 1:** Characteristics of study participants (60 patients)

**Variable**	
Age (years)	51 ± 13
Gender	
Men/Women (%)	44.2 / 55.7
Surgery	
CABG (%)	44.2
Replacement/ Valverepair (%)	55.7
BMI (Kg/m2)	24.8
ICU time (days)	3.5 ± 6
Time of hospital stay (days)	11.3 ± 6
Preoperative LV ejectionfraction (%)	60 ± 14
CPB time (min)	93 ± 27
FVC (Pre)	1.20 ± 0.4
FVC (Discharge)	1.63 ± 0.5
FVE1 (Pre)	2.02 ± 0.6
FVE1 (Discharge)	2.54 ± 0.8
NHP (Pre)	158.06 ± 99.01
NHP (Discharge)	160.70 ± 100
FIM (Pre)	123 ± 4
FIM (Discharge)	115 ± 10.8
DM (%)	20

BMI: body mass index, CPB: cardiopulmonary bypass, ICU: intensive care unit, LV: left ventricle; FVC - Forced Vital Capacity; FVE1 - Forced expiratory volume in one second; DM: diabetes mellitus. Data expressed in percentage of occurrence is the prevalence in this sample.

This study was approved by the Research Ethics Committee of the Federal University of Sergipe under protocol number CAAE-0180.0.107.107-11. All patients signed written informed consent before participation.

### Variables considered potentially associated with the 6MWD

Age, gender, type of surgery (coronary artery bypass grafting (CABG) or valve replacement (non-CABG)), days of hospitalization in the intensive care unit (ICU), length of hospitalization, duration of mechanical ventilation (MV), cardiopulmonary bypass (CPB) time, quality of life (Nottingham Health Profile, functional capacity (FIM), body mass index (BMI), preoperative left ventricular ejection fraction (LVEF), forced vital capacity (FVC), forced expiratory volume in one second (FEV1), presence of diabetes and hemoglobin dosage.

### The six-minute walk test (6MWT)

The 6MWT was performed according to American Thoracic Society [8]. A plan corridor with 30 m and free of obstacles was used. While the patient remained seated, we assessed respiratory rate, heart rate, blood pressure, peripheral oxygen saturation and subjective sensation of dyspnea (modified Borg scale) [8].

### Assessment of pulmonary function

Pulmonary function was characterized by spirometry using a portable spirometer manufactured by Clement Clarke ONE FLOW®. The forced vital capacity maneuvers were performed following the technical procedures, criteria for acceptability and reproducibility, as recommended by ATS [9].

### Functional capacity

Functional capacity was assessed by the Functional Independence Measure (FIM), which is part of the Uniform Data System for Medical Rehabilitation and has been widely used and accepted as a measure of functional assessment [10] and is validated in Brazil [11]. The FIM assesses the independence and assistance demanded by a person to perform a range of motor and cognitive tasks of daily living. Among the assessed activities we mention self-care, transfers, locomotion, sphincter control, communication and social interaction [10,11].

The FIM has 18 items of assessment and is composed of two fields: motor and cognitive. The motor field is composed of 13 items and divided into four categories: personal care, sphincter control, mobility/transfer, locomotion. The cognitive field consists of 5 items, in two categories: communication and social cognition. For each item is assigned a score ranging from 1 = full assistance, 2 = high assistance, 3 = moderate assistance, 4 = minimal assistance, 5 = supervision, 6 = modified independence and 7 = complete independence. Thus the total score ranges from 18 (total dependence) to 126 (complete independence) [11,12].

### Assessment of quality of life

To assess the quality of life we used the Nottingham Health Profile, a generic instrument originally developed to assess quality of life in patients with chronic diseases [13]. This is a self-administered questionnaire consisting of 38 items, based on the classification of disability described by the World Health Organization. Using a language easy to interpret, the NHP provides a simple measure of physical, social and emotional health of the individual being considered clinically valid for distinguishing patients with different levels of impairment and to detect significant changes in the framework of the patient over time [14].

### Data collected from the patient’s records

Type of surgery, duration of mechanical ventilation, CPB time, days of hospitalization, days of ICU admission, LVEF, hemoglobin and comorbidities (high blood pressure (hypertension)), diabetes and dyslipidemia).

### Statistical analysis

Data are presented as mean and standard deviation. We used univariate analysis to select the variables to be used in the multivariate analysis model. For univariate analysis, we consider a level of significance less than 20% (*P* < 0.20). In multivariate analysis, the non-significant variables were excluded from the model one at a time manually following the criterion of the highest *P* value. The model was determined when all the variables were presented with a level of significance less than 5% (*P* < 0.05). The dependent variable was the distance in the six-minute walk test. After the analysis, we establish an equation with the selected variables.

The variables considered for the univariate analysis were: age, gender, type of surgery (CABG or non-CABG), ICU days, days of hospitalization, duration of CPB, duration of MV, pre-LVEF, discharge NHP, discharge motor FIM, total discharge FIM, pre-FVC, discharge FVC, pre-FEV1, discharge FEV1, BMI, presence of diabetes and hemoglobin.

The reliability of our equation was assessed using the Bland and Altman representation for comparison of the measured and predicted distance with the equation proposed in a second group of 6 patients, 10% of the sample that generated the equation [15].

## Results

**Figure 1:**
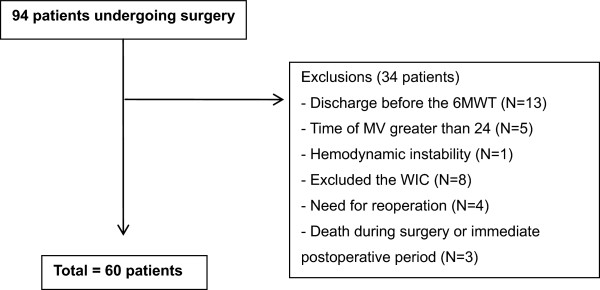
Flowchart of study patients.

Ninety-four patients undergoing cardiac surgery were selected, 34 were excluded due to discharge before the 6MWT, MV time greater than 24 hours, need for reoperation, death and denial (Figure 1). The characteristics of the 60 patients assessed are summarized in Table 1.

The 6MWT was well tolerated by all patients and no test was interrupted before completing 6 minutes. The mean 6MWD was 260.20 ± 89.20 meters.

Univariate analysis selected the following variables considered in the multivariate analysis: age (*P* = 0.001), type of surgery (*P* = 0.037), ICU days (*P* = 0.037), CPB time (*P* = 0.13), total FIM at discharge (*P* = 0.074), BMI (*P* = 0.20) and presence of diabetes (*P* = 0.20).

The following variables, age (*P* = 0.69), presence of diabetes (*P* = 0.64) and ICU days (*P* = 0.99) (in order of exclusion) were excluded from the multivariate analysis model.

Multivariate analysis selected the following variables: type of surgery (*P* = 0.001), CPB time (*P* = 0.001), total FIM (0.004) and BMI (0.007), considering r = 0.91 and r2 = 0.83 with *P* < 0.001.

The equation derived from the multivariate analysis was:

$$\begin{array}{ll} 6\mathrm{MWD}= & \mathrm{Surgery}\;\left(89.42\right)+\mathrm{CPB}\;\left(1.60\right)\\ & +\mathrm{total}\;\mathrm{FIM}\;\left(2.79\right)‒\mathrm{BMI}\;\left(7.53\right)‒127.90 \end{array}$$

*considering 6MWT distance in meters; surgery type: 1 to CABG and 2 to non-CABG; CPB time in minutes; BMI in kg/m^2^.

Given that the type of surgery was the variable that most influenced the distance covered on the 6MWT, we decided to compare the profile of our patients who had undergone CABG and those who had not undergone CABG. The CABG group had higher age (*P* < 0.0001) and a higher percentage of risk factors such as hypertension, diabetes, dyslipidemia, history of smoking and alcohol consumption (Table 2).

**Table 2:** Characteristics of study participants stratified on the type of surgery

	**CABG**	**Non-CABG**	***P***
Age	57.88 ± 8.7	45.66 ± 12.5	<0.0001*
Gender			
Men	51.8%	33.3%	
BMI	24.53 ± 6.8	25.24 ± 4.7	0.99
ICU time (days)	2.3 ± 0.4	2.8 ± 0.7	0.006*
Time of hospital stay (days)	10 ± 2.7	14.7 ± 10.97	0.12
Preoperative LVEF (%)	60.25 ± 13.0	62.54 ± 10.63	0.87
CPB time (mim)	98.33 ± 40.99	83.38 ± 28.47	0.1088
Time of MV (hours)	11.54 ± 3.5	11.04 ± 5.22	0.1945
Ex –smokers (%)	51.8	21.2	0.0005*
Drinker/Ex-drinker (%)	48.1	30.3	0.057
Diabetics (%)	40.7	3	<0.0001
HBP (%)	74	48.4	0.026
Dyslipidemia (%)	33.3	18.5	0.05

CABG: coronary artery bypass grafting, BMI: body mass index, CPB: cardiopulmonary bypass, ICU: intensive care unit, LVEF: left ventricular ejection fraction; MV: Mechanical ventilation; HBP: systemic arterial hypertension,T Test was performed for parametric variables, Mann–Whitney test for nonparametric variables and Chi-Squared test for categoric variables. (*P < 0.05). Data expressed in percentage of occurrence is the prevalence in this sample.

### Equation reliability

The mean 6MWD assessed in a second group of patients (n = 6), used to test the reliability of the equation generated was 330.14 ± 55.51 meters, which is 106 ± 13% of predicted value, calculated with our equation. The correlation between predicted and measured values was significant (r = 0.76,*P* < 0.05).

**Figure 2:**
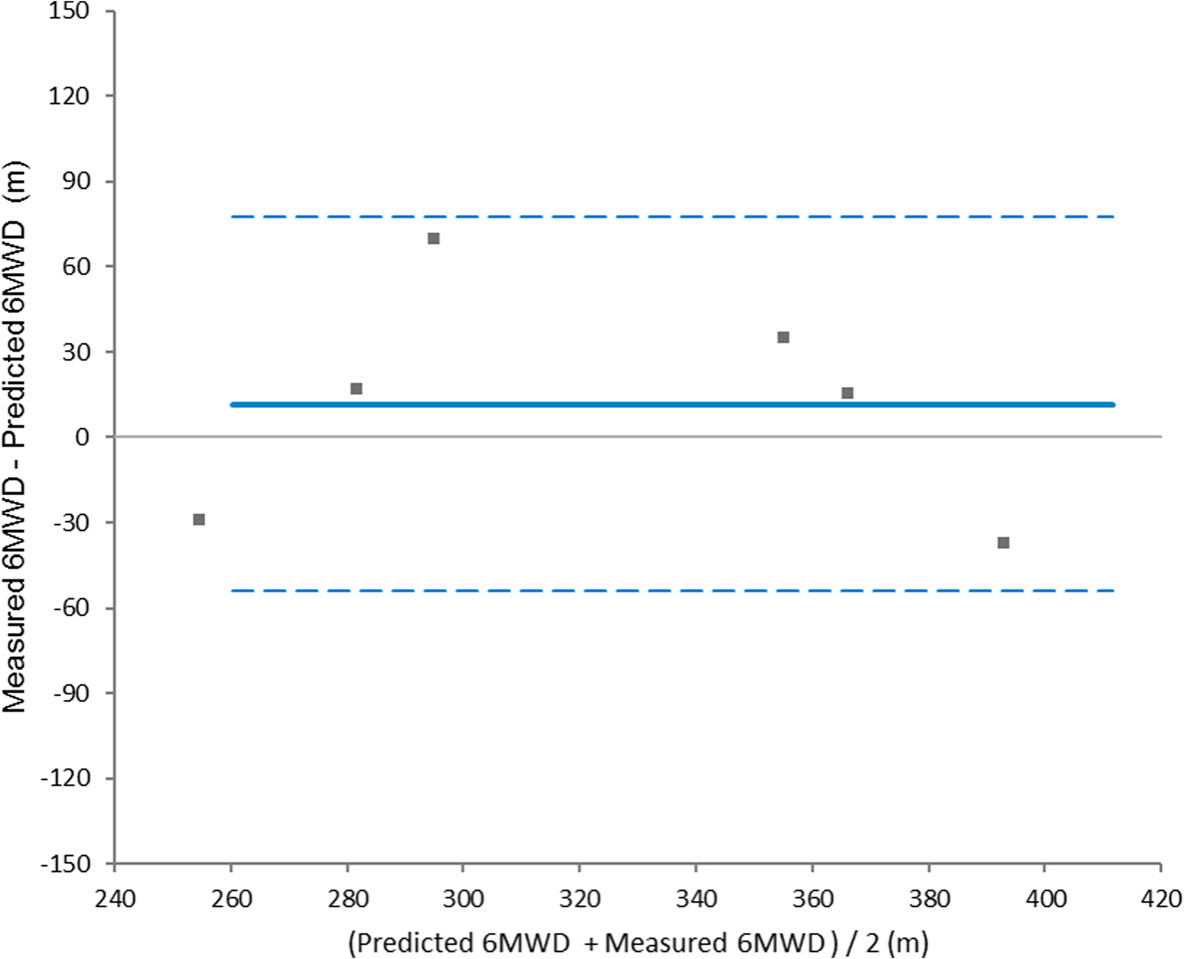
Representation of Bland and Altman Measure of the Walked Distance in the 6-minute Walk Test (6MWD) and the predicted distance by the proposed equation.

Figure 2 shows the Bland Altman [15] graphical representation of the comparison between the achieved 6MWT in the second group of patients and predicted 6MWD by the proposed equation.

## Discussion

The main finding of this study was that the 6MWD at hospital discharge in patients undergoing cardiac surgery was determined by the type of surgery (CABG or not), CPB time, total FIM and BMI.

Our study is the first to investigate the determinants of 6MWD in patients undergoing cardiac surgery using noninvasive and easy variables to be collected in clinical practice. Moreover, we were the first to propose and test a predictive 6MWD equation at hospital discharge in patients undergoing cardiac surgery.

In our study, we observed that the 6MWD was directly associated with the type of surgery, CPB and the Functional Independence Measure (FIM). Furthermore, the 6MWD was negatively associated with BMI.

Our results showed the type of surgery (CABG or non-CABG) as the main predictive variable for the 6MWD. The literature is controversial regarding the influence of the type of surgery and physical capacity in postoperative [6,16]. Our study, however, shows a better performance for patients undergoing non-CABG surgery. Studies show that individuals who exhibit a combination of cardiovascular risk factors have a poorer performance on the 6MWT compared to healthy individuals [17,18]. In general, in our study, it was observed that patients undergoing CABG showed a higher percentage of cardiovascular risk factors, which may indeed be negatively influencing walking ability in this group.

In our study, the influence of CPB in 6MWD was minimal, but positive, and this may have occurred because of the more severe patients were excluded from the group. There are no records in the studies assessing the influence of CPB on walking capacity literature, but some studies show that CPB does not influence the outcome of pulmonary function and postoperative gasometry [19,20].

The negative influence of BMI is well established in the field of exercise physiology. Enrigth, et al. [21]. noted that overweight may influence walking and increase the workload. Obesity increases the workload for a given amount of exercise, resulting in a lower 6MWD in patients with higher body weight or BMI.

The influence of gender on the 6MWD has been described in some studies [18,21,22], however in our study gender was not a predictive variable of the 6MWD in our population. The absence of gender as a predictor of 6MWD is consistent with observations from other studies [18,23] which showed that after adjusting for height and weight, there is no difference in MWD between men and women.

Other studies showed that spirometric values showed a strong positive correlation with the 6MWD in healthy adults [24,25] and in our study the pulmonary function variables (FVC and FVE1) were not represented in the final model, probably due to the homogeneity of the sample.

The combination of independent variables was able to explain 86% of the variability in 6MWD in the total sample, which is very satisfactory in comparison with previously published equations for healthy individuals. A previous study found models that explained 42% and 38% of the variation in 6MWD for healthy men and women, respectively [21]. Other factors that were not assessed in this study may, however, have influenced the 6MWD: percentage of lean body mass, quadriceps strength, leg length, level of physical activity prior to cardiac surgery [26,27].

### Limitation of the study

Our study has some potential limitations such as 6MWD assessment before surgery and did not include other variables such as the characterization of the physical activity level before surgery that could influence the functional status of patients. Moreover, the interpretation of our data should be cautious to represent patients from a cardiology service alone.

## Conclusion

In this study, the determinants of 6MWD at hospital discharge in patients undergoing cardiac surgery were: the type of surgery, CPB time and the Functional Independence Measure and body mass index. This study generated a reliable equation to predict 6MWD.

## Abbreviations

6MWD: Distance walked in six-minute walk test; 6MWT: Six-minute walk test; BMI: Body mass index; CABG: Coronary artery bypass grafting; CPB: Cardiopulmonary bypass; FEV1: Forced expiratory volume in one second; FIM: Functional independence measure; FVC: Forced vital capacity; ICU: Intensive care unit; LVEF: Left ventricular ejection fraction; MV: Mechanical ventilation; NHP: Nottingham health profile.

## Competing interests

The authors declare that they have no competing interests.

## Authors’ contributions

GUO, VOC, LPAC, AAAF, MLCN, WMSJ, TCFC and VJSF conceived and planned the activities that led to the study. GUO and LPAC performed assessments. GUO assessed the data. GUO, VOC, MLCN, WMSJ and VJSF prepared the manuscript. All authors read and approved the final manuscript.
